# Clinical and Biochemical Profiles of Hospitalized Patients with Hypercalcaemia from a Tertiary Care Centre in North India

**DOI:** 10.17925/EE.2024.20.2.15

**Published:** 2024-10-04

**Authors:** Aman Kumar, Deepak Khandelwal, Lovely Gupta, Upasana Agrawal, Suresh Mittal, Vivek Aggarwal, Nishikant Avinash Damle, Monika Garg, Deep Dutta, Sanjay Kalra

**Affiliations:** 1. Department of Medicine, Maharaja Agrasen Hospital, Punjabi Bagh, New Delhi, India; 2. Department of Endocrinology & Diabetes, Khandelwal Diabetes,Thyroid & Endocrinology Clinic, Paschim Vihar, New Delhi, India; 3. Department of Research, Sitaram Bhartia Institute of Science and Research, Qutab Institutional Area, New Delhi, India; 4. Department of Internal Medicine, LSU Health Sciences Center, Shreveport, LA, USA; 5. Department of Endocrine Surgery, Maharaja Agrasen Hospital, Punjabi Bagh, New Delhi, India; 6. Department of Nuclear Medicine, All India Institute of Medical Sciences (AIIMS), New Delhi, India; 7. Department of Radiology, Maharaja Agrasen Hospital, Punjabi Bagh, New Delhi, India; 8. Department of Endocrinology, Center for Endocrinology Diabetes Arthritis & Rheumatism (CEDAR) Superspeciality Clinics, Dwarka, New Delhi, India; 9. Department of Endocrinology, Bharti Hospital & BRIDE, Karnal, India

**Keywords:** Humoral hypercalcaemia, hypercalcaemia, multiple myeloma, parathyroid hormone, primary hyperparathyroidism, vitamin D toxicity

## Abstract

**Background and aims:** The profile of hypercalcaemia in hospitalized patients in India seems to be changing. However, studies evaluating the profile of hypercalcaemia in hospitalized settings in India are extremely limited. This prospective study aims to evaluate the clinical and biochemical profile of hospitalized patients with hypercalcaemia from a tertiary care centre in north India. **Materials and methods:** Clinical and biochemical profiles of subjects with hypercalcaemia detected during hospitalization/hospitalized with hypercalcaemia were assessed. A total of 91 subjects with sustained hypercalcaemia, who were eligible, underwent further investigation as per the institutional protocol and the data collected were analyzed. **Results:** The mean age of participants was 57.88 ± 14.23 years, with 62.64% of participants being females. The most common symptoms were nausea and anorexia, which were observed in all patients. The most common clinical sign was dehydration, which was observed in 32.97% of subjects. Primary hyperparathyroidism was the most common cause (41.76%), followed by suspected or confirmed malignancy/solid tumours in 15.38% of subjects. Other causes were advanced chronic liver disease (10.99%), multiple myeloma (9.89%), vitamin D toxicity (8.79%), granulomatous disorders (2.20%) and drug-i nduced disorders (1.10%). Forty-one subjects (45.05%) developed acute kidney injury and 14 subjects (15.38%) developed acute pancreatitis as a complication. Six subjects (6.59%) died during the course of hospitalization because of either primary disease or other secondary complications. **Conclusions:** Clinicians should be aware of changing patterns of hypercalcaemia in a hospital setting. Hypercalcaemia in hospitalized patients is associated with significant complications and mortality. Further large-scale prospective studies are needed to understand the changing pattern of hypercalcaemia in hospitalized patients from India.

Hypercalcaemia is a common clinical condition in hospitalized patients. Malignancies and primary hyperparathyroidism (PHPT) are the two most common causes of hypercalcaemia in hospitalized patients.^1–3^ Apparently, there is a changing profile of hypercalcaemia in India, especially in hospital settings, because of increasing reports of vitamin D toxicity and the early detection of PHPT, necessitating clinicians to be aware of such changes for effective diagnosis and treatment.^4–12^ The advent of rapid and reliable automated methods for measuring serum calcium makes it possible to detect hypercalcaemia early in the course of the disease, even when patients are asymptomatic or when patients are evaluated for unrelated conditions. Calcium estimations are made on routine blood analysis in almost every patient admitted to our hospital. Studies reporting the profile of hypercalcaemia in hospitalized patients from India are limited, and most are retrospective in nature.^4,13,14^ There are limited, prospective well-planned studies to investigate the profile of hypercalcaemia in hospitalized patients from India. This prospective study aims to evaluate the clinical and biochemical profiles of hospitalized patients with hypercalcaemia.

## Methods

### Study design

This study was conducted in a 300-bed teaching tertiary care hospital in New Delhi, India, serving patients from both urban and rural areas, between 1 April 2019 and 31 March 2020. The study was approved by the institutional ethics committee. All subjects with hypercalcaemia detected during hospitalization/hospitalized with hypercalcaemia were offered to participate in the study. Only those subjects who gave written informed consent were included in the study. In our laboratory, the reference range for serum calcium is between 8.5 and 10.5 mg/dL. The method used in our laboratory for calcium detection is the Arsenazo III method. Serum calcium was rechecked for the next 2 consecutive days in all subjects with the first report of hypercalcaemia by taking all due precautions such as samples without tourniquet application. Sustained hypercalcaemia was labelled if there were two or more readings of albumin-corrected total serum calcium levels above 10.5 mg/dL (an upper limit of the laboratory reference range in our hospital) at least 24 hours apart. Subjects with sustained hypercalcaemia were further evaluated and included in the analysis.

**Figure 1:**
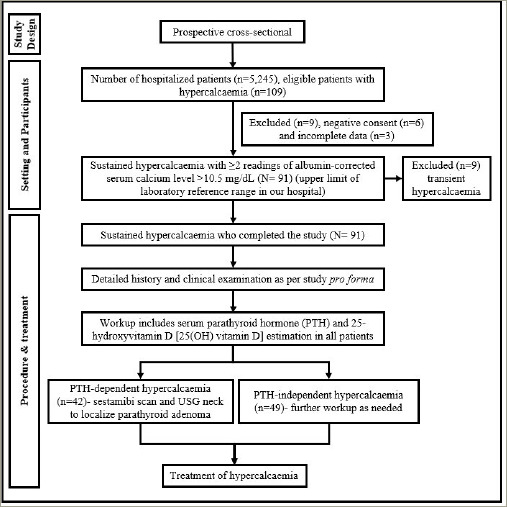
Study methodology flow chart

A detailed history and clinical examination were conducted as per the study *pro forma*. All workup for hypercalcaemia was conducted as part of routine clinical practice, according to standard clinical practice protocols. In our hospital, the protocol for sustained hypercalcaemia workup includes serum parathyroid hormone (PTH) and 25-hydroxyvitamin D (25(OH) vitamin D) estimation in all patients. Further investigations were carried out depending on the reports of PTH and 25(OH) vitamin D. Patients with elevated or inappropriately normal PTH in a setting of hypercalcaemia were classified as PTH-dependent hypercalcaemia, while patients with suppressed PTH in a setting of hypercalcaemia were classified as PTH-i ndependent hypercalcaemia. All subjects with PTH-dependent hypercalcaemia underwent sestamibi scan and neck ultrasonography (USG) to localize parathyroid adenoma. Neck USG was performed by a single experienced radiologist in our hospital. Vitamin D toxicity was defined as serum 25(OH) vitamin D levels ≥100 ng/mL (≥250 nmol/L), along with suppressed PTH. Workup for multiple myeloma and malignancies was conducted for PTH-i ndependent hypercalcaemia if needed. The diagnosis of multiple myeloma was based on immunofixation or bone marrow aspiration studies. Patients with diagnosed/suspected malignancies were re-referred to other oncology hospitals for further management, as an oncology facility was not available in our hospital. Treatment of hypercalcaemia was performed in line with the standard clinical protocol as decided by the treating physician, and information pertaining to that was also captured. The flow chart of the study methodology is represented in Figure 1.

### Statistical analysis

The categorical variables were presented as numbers and percentages (%) and the continuous variables as mean ± standard deviation (SD) and median values. The data entry was performed in a Microsoft Excel spreadsheet and the final analysis was conducted using Statistical Package for Social Sciences (SPSS) software version 21.0.

## Results

We identified 109 subjects with hypercalcaemia among the total number of 5,245 patients admitted to our hospital during the study period. Six subjects did not give consent for the study and complete data for 3 subjects were not available for the analysis; hence, these nine subjects were excluded. Out of the remaining 100 subjects, 9 subjects showed transient hypercalcaemia, and the remaining 91 subjects had sustained hypercalcaemia. These 91 patients were further worked up in detail, and their data were analyzed. The mean age (years ± SD) of the subjects under study was 57.88 ± 14.23 years. Fifty-seven subjects (62.64%) were female and 34 subjects (37.36%) were male. Forty-four subjects (48.35%) had diabetes and 45 subjects (49.45%) had a history of hypertension. Corrected total serum calcium at the time of presentation showed a mean of 12.17 ± 1.12 mg/dL (ranging from 10.70 to 16.50 mg/dL;*Table 1*). Other biochemical parameters of the study subjects are summarized in *Table 1*. The median serum 25(OH) vitamin D levels of the study subjects were 78.50 nmol/L (ranging from 14.00 to 471.60 nmol/L). Vitamin D was deficient in 22 (24.18%), normal in 61 (67.03%) and in the toxic range in 8 (8.79%) subjects.

*PTH = parathyroid hormone; USG = ultrasonography.*

**Table 1:** Biochemical parameters

Parameter (normal range)	Mean ± SD or median (range)
Serum total calcium (albumin-corrected) at presentation (8.5–10.5 mg/dL)	12.17 ± 1.12
Serum albumin (3.5–4.7 g/dL)	3.75 ± 0.35
Serum phosphate (2.3–4.7 mg/dL)	3.88 ± 1.81
Serum ALP	281.33 (86.00–
(40–150 U/L)	989.00)
Serum PTH level (15–65 pg/mL)	150.03 (12.30–1739.40)
Serum 25(OH) D (30–100 nmol/L)	78.50 (14.00–471.60)

*N=91.*

*ALP = alkaline phosphate; 25(OH) D = 25-hydroxyvitamin D; PTH = parathyroid hormone; SD = standard deviation.*

Nausea and anorexia were the most common symptoms of hypercalcaemia, which were experienced by all study subjects. Clinically, dehydration was observed in 30 subjects (32.97%;*Table 2*). Ten subjects (10.98%) had a history of renal calculi. Of the total study subjects, 49 subjects (53.84%) were PTH-i ndependent and 42 subjects (46.16%) were PTH-dependent. Of the 42 subjects with PTH-dependent hypercalcaemia, 38 subjects were diagnosed with solitary parathyroid adenoma on sestamibi scan and neck USG. Of those having parathyroid adenoma, 10 underwent surgical parathyroidectomy after stabilization during current hospitalization in discussion with the patient and their family. Others were medically managed and advised for surgery subsequently.

**Table 2:** Clinical symptoms and signs

Symptom or sign	n (%)
Nausea	91 (100.00)
Anorexia	91 (100.00)
Vomiting	59 (64.83)
Abdominal pain	50 (54.95)
Constipation	49 (53.85)
Fatigue	37 (40.66)
Dyspepsia	31 (34.06)
Dehydration	30 (32.97)
Polyuria	13 (14.28)
Bony pain	11 (12.09)
Bony tenderness	7 (7.69)
Weight loss	6 (6.59)
Depression	6 (6.59)
Confusion/altered sensorium	6 (6.59)
Proximal muscle weakness	2 (2.20)

*N=91.*

**Table 3:** Distribution of aetiology of hypercalcaemia

Final diagnosis (aetiology)	n (%)
Primary hyperparathyroidism with solitary parathyroid adenoma	38 (41.76)
Suspected/confirmed malignancy/solid tumours	14 (15.38)
Chronic liver disease	10 (10.99)
Multiple myeloma	9 (9.89)
Vitamin D toxicity	8 (8.79)
Granulomatous disorders	2 (2.20)
Drug-i nduced disorders	1 (1.10)
Others (undiagnosed)	9 (9.89)

*N=91.*

Fourteen (15.38%) subjects either had confirmed or suspected malignancy (solid tumours); after the initial stabilization, these subjects were referred to other oncology centres, as the oncology facility was not available in our hospital (*Table 3*). Multiple myeloma was diagnosed in another nine subjects; again, these were referred to oncology centres for further management. Ten (10.99%) subjects had hypercalcaemia related to advanced chronic liver disease (CLD); they had no other causes of hypercalcaemia except for advanced CLD. Eight (8.79%) subjects had vitamin D toxicity-related hypercalcaemia; all of these subjects had suppressed PTH. In addition, other causes of hypercalcaemia, especially malignancy, were excluded from these subjects. Of the two subjects with granulomatous disease, one subject was diagnosed with tuberculosis and another with sarcoidosis. One subject had chlorthalidone (thiazide)-induced hypercalcaemia, which normalized after stopping the drug. In nine subjects (9.89%), the cause of hypercalcaemia could not be ascertained.

For treatment, intravenous fluids were given to all subjects, loop diuretics to 90 (98.9%) subjects, subcutaneous calcitonin to 25 (27.47%) subjects, intravenous bisphosphonates to 5 (5.49%) subjects and steroids to 7 (7.69%) subjects. Haemodialysis was required in 19 (20.88%) subjects for the management of hypercalcaemia. Forty-one subjects (45.05%) developed acute kidney injury (AKI), and 14 subjects (15.38%) developed acute pancreatitis as a complication of hypercalcaemia. Six subjects (6.59%) died during the course of hospitalization because of either primary disease or other secondary complications. The mean duration of hospital stay for the entire study participants was 6.62 ± 2.10 days. The mean duration of hospital stay in participants with vitamin D toxicity was significantly longer compared with the rest of the participants (8.6 ± 1.30 versus 6.44 ± 2.07 days; p<0.01). Except for one subject, all other patients had normal serum calcium at the time of discharge. The mean corrected total serum calcium on discharge in subjects who were discharged was 9.09 ± 0.53 mg/dL (ranging from 8.2 to 10.5 mg/dL).

## Discussion

In the current study, we looked into the aetiology and clinical and biochemical profiles of hospitalized patients with hypercalcaemia and have found changing profiles of hypercalcaemia in hospitalized patients. Extremely limited prospective studies have investigated the clinical and biochemical profiles of hospitalized patients with hypercalcaemia from India.^15^

The clinical symptoms and signs were, in general, consistent with the published literature.^1,16^ The study subjects showed nausea and anorexia as the leading symptoms, followed by vomiting, abdominal pain, constipation and fatigue. There was a decline in the number of patients having a history of renal calculi in our study. The reduced presentation of bone disorder and renal stone is consistent with recent Indian studies, which draw a shift in the presentation of hypercalcaemia as asymptomatic due to the earlier detection. The biochemical profile of the subjects was also consistent with previous studies.^1,16^

**Table 4:** Published Indian studies on hypercalcaemia in hospitalized patients^4,14,15,21^

Study (year)	Design of the study	Total patients studied	Key findings
Sukhija et al. (2023)^21^	Prospective	200	Malignancy was the most common cause, followed by PHPT and drug-i nduced hypercalcaemia No significant correlation between the cause of hypercalcaemia and hospitalization outcome
Sulaiman et al. (2022)^15^	Prospective	150	Malignancy was the most common cause (41.3%), followed by PHPT (32.7%), vitamin D toxicity (8.7%), advanced CLD (2.7%) and indefinite aetiology in 7.3% of cases Cumulative mortality rate was 28% at the end of 6 months
Kuchay et al. (2017)^4^	Retrospective	552	Malignancy was the most common cause (23.1%), followed by PHPT (21.9%), advanced CLD (8.9%) and vitamin D toxicity (5.5%)
Gupta (1990)^14^	Retrospective	89	Malignancy was the most common cause (80.9%), followed by PHPT (12.4%), hypervitaminosis D (5.6%) and sarcoidosis (1.1%)

*CLD = chronic liver disease; PHPT = primary hyperparathyroidism.*

In our study, the most common cause of hypercalcaemia was PHPT with localized parathyroid adenoma (41.76%). A retrospective study conducted at another tertiary care centre from India by Kuchay et al. demonstrated that PHPT was the cause of hypercalcaemia in 21.9% of hospitalized subjects, which was almost comparable with malignancy (23.1%).^4^ In our study, 15.38% of cases of hypercalcaemia were because of suspected/confirmed solid tumours, and another 9.89% were diagnosed with multiple myeloma. Hypercalcaemia occurs in up to 30% of patients with malignancy and is the most common group of hypercalcaemia in the hospital setting in most series.^17,18^ The malignancies most associated with hypercalcaemia are epidermoid malignancies (squamous cell lung carcinoma, urothelial cancers and head and neck cancers), multiple myeloma, breast cancer, renal cell carcinoma and lymphoma.^19,20^ In our study, malignancies accounted for relatively fewer cases of hypercalcaemia; one of the reasons was the absence of oncology facilities in our hospital. In*Table 4*, we summarize all the previously published studies of hypercalcaemia in hospitalized patients from India.^4,14,15,21^

One of the highly important causes of hypercalcaemia in the present study was advanced CLD, contributing to 10.99% of cases. A considerable proportion of subjects with decompensated CLD develop hypercalcaemia during the course of the disease.^22^ Gerhardt et al. described the same pattern of hypercalcaemia in 11 patients who had advanced CLD but no hepatic cancer.^21^ Hypercalcaemia caused by advanced CLD without hepatic neoplasia is a poorly understood condition. The unique feature of this type of hypercalcaemia is its transient nature, which may or may not require treatment. This group of non-parathyroid hypercalcaemia is now commonly recognized in any tertiary care centre where there is an advanced hepatology unit.^22,23^

Hypercalcaemia secondary to hypervitaminosis D is another common cause of hypercalcaemia observed nowadays. This entity was considered rare a few decades ago. Overzealous supplementation of vitamin D in individuals without metabolic bone disease has led to the emergence of an increasing number of cases of vitamin D toxicity in developing countries, where vitamin D supplements, in general, are available over-the-counter, and injectable vitamin D preparations are often used irrationally.^5–7^ A prospective, random-sample study conducted in India in 2017 found that vitamin D intoxication, primarily caused by excessive pharmaceutical use, is the most common cause of hypercalcaemia.^24^ In the present study, a total of eight (8.79%) patients had hypercalcaemia related to vitamin D toxicity. Khan et al. in a study from a tertiary care centre in Pakistan found vitamin D toxicity as the second most common cause of hypercalcemia after PHPT.^25^ The increase in hypervitaminosis D has raised concerns about the injudicious use of vitamin D supplements.^26^

In our study, one subject had tuberculosis and another had sarcoidosis. Harrell and Fisher established the association of hypercalcaemia and granulomatous disease in 1939.^27^ Various granulomatous diseases associated with hypercalcaemia are sarcoidosis, tuberculosis, leprosy, berylliosis and disseminated candidiasis.^3^ These granulomatous disorders, especially infective disorders, still contribute to hypercalcaemia cases in South Asian countries.

In our study, AKI (45.05%) and acute pancreatitis (15.38%) were the leading complications of hypercalcaemia with high mortality during the hospital course (6.59%) because of either primary disease or other secondary complications. A prospective observational study conducted in north India showed that hypercalcaemia was prevalent in 1.5% of Asian-I ndian patients admitted to a tertiary care hospital. A cumulative mortality rate of 28% was observed, with underlying malignancy and serum calcium levels being independent predictors.^15^ A recently conducted longitudinal, retrospective multicentre study from Malaga in 2023 similarly showed that hypercalcaemia in hospitalized patients is primarily caused by neoplastic processes (75.1%), as well as PHPT and medications (both 8.8%), leading to high mortality rates (81.5%) accelerated by advanced age, neoplastic aetiology and medication failure. Another retrospective study from Switzerland by Ravioli et al. showed malignancy as the most common cause of severe hypercalcaemia and 16% mortality during the course of hospitalization in these patients with severe hypercalcaemia.^28^ This highlights the need for a multidisciplinary approach with customized management plans for hypercalcaemia, involving comprehensive profiling, laboratory tests, imaging studies and specialized assays for optimal diagnosis, treatment and improved patient outcomes.^21,29^

The major strengths of this study were its prospective design, a systematic delineation of the aetiologies of hypercalcaemia and the inclusion of clinically relevant patient outcomes, such as the development of AKI, acute pancreatitis and mortality. This study is a good addition to the limited Indian literature on this subject. A few limitations of our study need to be mentioned. The small sample size of this study, which is also from a single tertiary care centre, limits the generalizability of the study findings. In addition, the non-facility of oncology services at our centre may result in low cases of malignancy-related hypercalcaemia in this study. Future research should involve multicentric studies, involving diverse populations from various regions and hospitals, especially those with medicine/endocrinology and oncology departments, for better representation.

## Conclusion

Clinicians should be aware of the changing pattern of hypercalcaemia in the hospital setting. Early detection of PHPT is common, and as observed in our study, it can now be the most common cause of hypercalcaemia in hospitalized patients, especially in non-oncology setups. Vitamin D toxicity is common these days, especially in developing nations because of overzealous supplementation and injudicious use of high-dose injectable vitamin D preparations. Furthermore, the poorly reported hypercalcaemia of advanced CLD is observed frequently in tertiary care hospitals with a large volume of gastroenterological services. Chronic infective granulomatous diseases, such as tuberculosis, still contribute to the non-parathyroid group of hypercalcaemia in South Asian countries. Hypercalcaemia still contributes to significant morbidity and mortality in hospitalized patients.
